# Comradeship in Hospital Life

**Published:** 1920-03-27

**Authors:** 


### Comradeship in Hospital Life.

Most of the lay employees of the Royal Victoria Infirmary, Newcastle-upon-Tyne, served either in the Navy or the Army during the war. On their return they sought to introduce into the daily doutine of civil life that comradeship which was the life of the fighting forces. With that object in view they formed an Employees' Social Committee, under the chairmanship of Mr. W. G. Nevin. This Committee arranged a variety of social gatherings, all of which have been much appreciated. Mr. Orde, the House Governor and Secretary, having entered cordially into the spirit of these functions, made it a point of being present on each ocacsion. The winter series was brought to a conclusion on March 18, 1920, by a whist drive and social, held in the Drawing-room Café, Newcastle.

During the course of the evening Mr. Nevin referred to the charity ball which his Committee had organised last January, when over 380 people attended. Though the tickets were only 5s. each, they had in hand, after paying all expenses, the sum of £61 5s., which they desired to devote to the Special Appeal Fund of the Royal Victoria Infirmary. In reply, Mr. Orde expressed the pride he felt in having such a loyal band of employees assisting him in carrying on the work of the hospital. It would give him the greatest pleasure to hand over their splendid gift to the House Committee. He knew it was a gift disinterestedly given, and set a worthy example for others to follow.

